# “You Can’t Go to the Park, You Can’t Go Here, You Can’t Go There”: Exploring Parental Experiences of COVID-19 and Its Impact on Their Children’s Movement Behaviours

**DOI:** 10.3390/children8030219

**Published:** 2021-03-12

**Authors:** Negin A. Riazi, Kelly Wunderlich, Madelaine Gierc, Mariana Brussoni, Sarah A. Moore, Mark S. Tremblay, Guy Faulkner

**Affiliations:** 1 School of Kinesiology, The University of British Columbia, 210-6081 University Boulevard, Vancouver, BC V6T 1Z1, Canada; negin.riazi@ubc.ca (N.A.R.); kelly.wunderlich@ubc.ca (K.W.); madelaine.gierc@ubc.ca (M.G.); 2 Department of Pediatrics, The University of British Columbia, Rm 2D19, 4480 Oak Street, Vancouver, BC V6H 3V4, Canada; mbrussoni@bcchr.ubc.ca; 3 School of Health and Human Performance, Dalhousie University, Stairs House, 6230 South Street, Halifax, NS B3H 1T8, Canada; sarah.moore@dal.ca; 4 Healthy Active Living and Obesity Research Group, Children’s Hospital of Eastern Ontario Research Institute, 401 Smyth Road, Research Building 1, Ottawa, ON K1H 8L1, Canada; mtremblay@cheo.on.ca

**Keywords:** physical activity, screen time, qualitative, outdoor play, interview

## Abstract

The COVID-19 outbreak and related public health guidelines have changed the daily lives of Canadians and restricted opportunities for healthy movement behaviours for children. The purpose of this study was to explore how parents experienced the pandemic-related restrictions and how they impacted their children’s movement behaviours. **Methods**: Twenty-nine semi-structured one-on-one interviews were conducted (June–July 2020) with parents of children (5–11 years old) in Ontario and British Columbia. Interviews lasted between 24–104 min, were audio-recorded, transcribed verbatim, and thematically analyzed. **Results**: Findings emphasized various individual (e.g., motivation), interpersonal (e.g., parent work schedule), built (e.g., closure of parks) and natural environment (e.g., weather) factors related to children’s movement behaviours. The findings highlighted the loss of structured activities and destinations for children’s physical activity, and restricted opportunities for outdoor play exacerbated by shrinking childhood independent mobility. **Conclusion**: Families are adapting to many pandemic-related challenges including adhering to public health restrictions, parents juggling multiple roles, conducting work and school from home, as well as exacerbating factors like weather. It will be important to continue to encourage outdoor time, support policies and practice that facilitate independent mobility, and develop centralized resources that help families in the maintenance of healthy movement behaviours.

## 1. Introduction

Habitual healthy movement behaviours are important contributors to child mental and physical health. Canadian 24-hour movement guidelines recommend that children aged 5–11 years achieve at least 60 min of moderate-to-vigorous physical activity (MVPA) per day; engage in several hours of light physical activity per day; sleep for 9–11 h per night; and have no more than 2 h per day of recreational screen time (ST). Children who meet these recommendations have better cardiometabolic, musculoskeletal, cognitive, mental health, and immune function, on average, compared with those who do not [1].

Despite the clear health benefits of these movement behaviours, approximately 30% of Canadian children 5–11 years meet the 24-Hour Movement Guidelines [2]. The COVID-19 pandemic has inflicted further collateral damage on child health through a range of lockdown restrictions. The closure of schools, playgrounds, recreational facilities and in some communities, public parks/places restricted opportunities to be outdoors and physically active. In Canada, a cross-sectional study of 1472 parents of children aged 5–11 years found that only 4.8% of participants met the combined 24-hour movement guidelines [3]. Children were reported as having lower physical activity (PA) levels, less outside time, higher sedentary behaviour (including increased leisure ST), and more sleep during the pandemic.

These data further highlighted that the impact of pandemic-related restrictions were not uniform for Canadian children [3]. Mitra and colleagues (2020) identified that children decreased walking/biking and outdoor play after COVID-19 related restrictions were imposed, with children living in neighbourhoods with higher dwelling density and closer to major roads being less likely to increase outdoor activities [4]. de Lannoy and colleagues (2020) explored regional variations in change in time spent outdoors and in outdoor play [5]. While there was a decrease overall in all regions, Ontario experienced the greatest decline in children’s time spent outdoors and in outdoor play compared to other provinces. Provinces that have had the highest number of COVID-19 cases in Canada, such as Ontario and Québec, have also experienced the most stringent restrictions on access to the outdoors.

Additionally, previous research has underscored the importance of parenting practices and children’s physical activity. Jago et al. found that higher maternal and paternal logistic support (e.g., providing transportation to parks and playgrounds, enrolling child in activities) was related to greater physical activity [6]. Children engaged in more physical activity when they had parental logistic support and permissive parents (compared to authoritative parents) [6]. Other research has shown that parental support is an important factor influencing youth physical activity [7]. However, no qualitative research has examined how the pandemic has impacted parental support of their child’s participation in the healthy movement behaviours of sleep, sedentary behavior and physical activity.

To extend and contextualize the cross-sectional study first reported by Moore and colleagues [3], the purpose of this study was to use qualitative methods to explore how parents experienced the pandemic and its related restrictions, and how those restrictions impacted the movement behaviours of their child(ren) (aged 5–11 years), taking into account factors that may explain potential variation (e.g., provincial or regional restrictions). Given the ongoing nature of the pandemic there is a time-sensitive need to understand how to help families preserve and promote child health during the COVID-19 crisis and recovery period and inform strategies to mitigate potential harm during future pandemics.

## 2. Materials and Methods

### 2.1. Design

Parents participated in semi-structured interviews about their children’s daily movement behaviours and outdoor play during the early stages (March–May 2020) of the COVID-19 pandemic and the impact of the pandemic and related-restrictions on these movement behaviours. This study was approved by the University of British Columbia’s Behavioural Research Ethics Board (#H20-01554).

### 2.2. Participants and Recruitment

Parents who participated in the national cross-sectional survey of parents of children [3] and who expressed interest in participating in an interview at the end of the survey were eligible to participate if they met the following inclusion criteria: (a) being a parent or guardian (“parent”) of a child aged 5–11 years; (b) living in either Toronto, Ontario or Vancouver, British Columbia; (c) able to understand and communicate in English; and (d) having access to a computer with the capability to access Zoom and with a reliable internet connection. The online survey was conducted by a third-party market research company (Maru) (see [3] for more details about recruitment). Participants who met the criteria were selected randomly by the third-party Maru. Participants were sent two follow-up e-mails; if they declined to participate and/or did not respond within 1 week, a new potential participant was randomly selected and contacted. Research aimed to recruit 15 participants from Toronto, Ontario and 15 participants from Vancouver, British Columbia, for a total of 30 participants; with the option of increasing sample size until data saturation was achieved [8]. These locations were chosen as regional differences were found in the cross-sectional study [5] and they were both large cities from where Maru typically recruits. Interactions were limited to emails to invite participation and arrange an interview, the interview, and a follow up email with a $50 e-gift card for participating. The final sample draws on data from 29 parents (BC, *n* = 12; ON, *n* = 17; see Table 1).

### 2.3. Measures

Sociodemographic information. Demographic information for participants who consented to participate was provided by Maru. Descriptive information collected included age, gender, ethnicity, marital status, education, household income, and children’s demographics (e.g., age, gender) (see Table 1).

Interview Guide. Drawing on a social-ecological framework that recognizes multiple layers of influence on behavior (e.g., individual, social, environmental, policy) [9], the interview guide was developed by the authors to explore changes in movement behaviours due to the pandemic, parental approaches to supporting healthy movement and outdoor play behaviours prior to and during COVID-19 pandemic (e.g., What has worked in supporting your child to engage in healthy movement behaviours?), pandemic-related restrictions (e.g., Having experienced the impact of physical distancing and other related restrictions, is there anything that would have helped you in supporting your child’s movement behaviours?), and existing and anticipated barriers and facilitators to movement and outdoor play behaviours. Questions were open-ended, such as “Let’s think about a typical day when you felt the restrictions were most challenging. Can you walk me through what that day was like for your child?” followed by prompts about different movement behaviours (i.e., physical activities, sedentary behaviours, sleep) and if or how they had changed. The interview guide can be found in Supplementary File S1.

### 2.4. Procedure

The current study adopted a similar qualitative methodology used in previous research (e.g., [10,11]). Semi-structured one-on-one interviews were conducted using Zoom (Zoom Video Communications; a videotelephony software) by MG and KW in June-July 2020, with interviews lasting 24–104 min (mean time: 60 min). Both interviewers have received training in qualitative methodology, have conducted many interviews in different research projects, and rehearsed the interview with each other before proceeding. Semi-structured interviews allowed for flexibility, producing rich and detailed data about participant experiences and perspectives [12]. Participants were assigned pseudonyms and were labelled by province (i.e., British Columbia = BC, Ontario = ON).

### 2.5. Analysis

Analysis of the transcribed documents followed an established protocol for thematic analysis [13,14]. Inductive thematic analysis was chosen given it is a flexible approach that can be used across a range of epistemologies and research questions [12]. The authors immersed themselves in the data by reading and re-reading the transcripts and discussions centered on examining facilitators and barriers for each movement behaviour (e.g., physical activity, sedentary behaviour). A range of strategies were implemented to enhance the “trustworthiness” of this study. Coding was done independently by two research members. Critical friends were employed in the analysis process in order to encourage “critical dialogue” and discussion around interpretations of the data and candidate themes (p. 113) [15]. Interpretations were shared and discussed with additional research team members to challenge identified themes and their connections. Coding was facilitated using Microsoft Excel 2016 where descriptive codes were created next to the related quotes to keep the meaning in context, and then grouped thematically.

## 3. Results

The results first present an overview of changes families faced early on in the pandemic and then describe three major themes common to all families including children’s (1) loss of structured activities and destinations for PA, (2) limited opportunities for outdoor play, and (3) pandemic-related rises in ST. Despite differences in living locations, overall families’ experiences were quite similar. Additionally, parents reported few changes in their child’s duration of sleep or quality of sleep.

### 3.1. “We Were Hit Hard from the Get-Go”: Stress and Change during the Early Stages of the COVID-19 Pandemic

Recounting the first three months of the pandemic, parents discussed many challenges their families faced including how the implementation of public health restrictions (e.g., stay-at-home, keep 2 metres apart, school closures) made the situation “feel more challenging” (Margaret, BC) and increased a sense of fear. Dakari (ON) explained that there was a fear “…of going out, afraid of talking to people…” Beside the need to “adapt really really quick” (Amelia, BC) to the rapidly evolving situation, most parents expressed concerns about children adhering to public health protocols. Braeden (ON) explained that “in terms of the physical distancing. It is hard for them [his children] to understand it and do it.” As a result of this concern, another parent explained she would not take her son with her to the grocery store “because he couldn’t not touch stuff” (Margaret, BC).

Other challenges for families included juggling children’s school, work, and increased stress related to these new circumstances. Balancing the transition to working-from-home and providing online schooling was most challenging. Robert (ON) explained that as a single parent he had “to work. On top of that now it requires me to sit down with [my daughter] and do the schoolwork. It was a handful. It took a lot of adjustment.” Some parents like Fernando (ON), had to be physically present at their jobs. He explained that he “[could not] work from home because [he] work[ed] in a hospital” and therefore, “had to go to work every day.” Other families spoke about the difficulty in helping their children with schoolwork especially with more than one child in the household. Amelia (BC), a mother of three, described having to tell her son’s school,

“I can’t do it [schoolwork with her child], I can’t, I’ve got the other two here. It would be one thing if it was one kid, but you know, you’ve got the other two crawling all over you and taking over and you know I’m still nursing one of them.”

Many families experienced stress related to children adjusting to online school, parents managing multiple roles, and families adjusting to new public health protocols. Additionally, families reported changes in their children’s movement behaviours related to physical activity, outdoor play, and screen time.

### 3.2. “Kids These Days Are Very Programmed”: The Loss of Structured Activities and Destinations for Physical Activity

With the pandemic-related restrictions instituted across the country, many families experienced a loss of PA opportunities, destinations and social activities with friends and family. As parents noted, much of their children’s pre-pandemic PA was accumulated through structured activities, and with closures of activities and destinations, families saw a dramatic decline in children’s PA. Major pandemic-related changes discussed by participants included a loss of structured activities at school and afterschool, limited access to destinations (e.g., parks), as well as various barriers (e.g., weather, motivation) that made it challenging to maintain previous PA levels. Parents also discussed not having enough time (e.g., work, schoolwork taking precedence), resources, or options to provide more PA opportunities for their children. Matthew (ON) explained that:

“We just don’t have the time or the resources to do it because I’m working full time, wife’s working full time. Once the weekends come around, the house has to be looked after and chores and this or that and by time we have some free time it’s just okay well, he’s playing Fortnite or the day’s done. It’s tough.”

This again highlighted the multiple roles parents were juggling during the pandemic.

There were various efforts to adapt to the situation. Some schools substituted physical education classes and recess by providing fitness-related activities (e.g., squats, push-ups), with this information sent as either written instructions, online pre-recorded videos, or live video-based physical education classes. Henry (ON) described that his daughter had “gym through the Google Meets twice a week through a 7-day cycle...They required 20-30 min of activity. She preferred sports, but we could not really accommodate.” While another parent, Fernando (ON), said “Well, they suggest that they go out for a walk, things like that. But otherwise, there is no formal class that they’ve started, no.” Many parents admitted that these online and fitness-based activities were uninteresting for children and did not provide the same intensity of activity that children used to get in physical education or recess. Brian (ON) confessed, “I guess it’s hard to get motivation because there’s no-one else around doing the same things,” referencing the fact that his child completed the PAs solo at home.

Most families described the loss of structured activities including organized sports. As Jessie (ON) explained, “Kids these days are very programmed…Every weekend was skating, gymnastics, dance or swim lessons and everything was pulled”—highlighting a prevalent reality for most families who lost access to the structured, extracurricular PA their child engaged in pre-pandemic. Ravi (ON) explained that his daughter began taking Zoom martial arts sessions “three times at least 40 min a session.” Another Ontario parent explained that his son was a competitive gymnast and practiced “nine hours a week”, but with the restrictions and closures, “It’s [PA] just totally, totally declined and there’s nothing, it’s just extremely difficult to replace that” (Matthew, ON). While some parents attempted to fill the void by providing “training at home” (Matthew, ON) and finding new ways to adapt (e.g., shifting from team games to individual skills), some children were not interested in the new adaptations. For example, while Carmen (BC) “tried to get the eight-year-old to do soccer drill and he’s like ‘nah’. He just doesn’t have any interest in it. He just didn’t want to do it.” To adapt, children were forced to practice alone, parents became coaches, or children missed out completely.

Families also attempted to address their children’s declining PA by incorporating other types of activities (e.g., Zoom classes, nature walks, bike riding, hikes). Other parents incorporated less structured activities like walking and cycling with their children, either scheduled at a certain time of day or whenever parents’ schedule would allow. Marcus (ON) said he “just taught [his son] how to ride [his bike] on his own without training wheels. That is all the physical activity he gets nowadays.” Moreover, some parents discussed the decline in children’s MVPA. As Dina (ON) explained,

“[Her family tries] to get outside and there’s still activity, it’s probably not really that racing heart, getting sweaty, really intense physical activity. It’s more we’ll go for a walk to the park, well we walk through the park, we don’t stop at the park [due to COVID-19 restrictions].”

### 3.3. “You Can’t Go to the Park, You Can’t Go Here, You Can’t Go There”: Limited Opportunities for Outdoor Play

With the closure of structured PA settings and activities (e.g., parks, playgrounds), outdoor spaces that were once available to families were suddenly inaccessible. Sanjay’s (ON) family lost access to their condominium’s facilities, like “the pool. There is a children[‘s] playground as well…All those are restricted. There were social distancing rules…going out to the malls and public parks and all those have been restricted for those three months.” The closures of many destinations created difficulties for families to find spaces where children could be physically active. Many parents described that they valued PA and tried to establish routines with children to incorporate PA, especially outdoors, into their new pandemic schedules. While some children wanted to be outside “even if it is cold or rainy or sunny or whatever” (Ravi, ON), other children were uninterested in going outside whether due to weather, playing outside on their own, having limited space (e.g., no backyard, park) or activities they could engage in, or being nervous about other people and being exposed to COVID-19. Brian (ON) clarified that his daughter had “never been an outdoorsy person so that makes it difficult…she’s a little bit frightened obviously because there’s, we’re on a very busy street and she’ll see people walking by so she’s nervous.”

Most parents did not grant their child(ren) the freedom to travel or play outside without supervision (i.e., children’s independent mobility). Several reasons for lack of children’s independent mobility included concern over traffic danger and adhering to physical distancing. Carmen (BC) described feeling that with no supervision, “…they might not do such a great job of physical distancing.” Much of the outdoor time that children experienced was contingent on parents’ schedules. As two parents put it, working from home, “every day is a working day” (Ravi, ON) so activities have to be scheduled after work or “just finding the time to do it, between our own schedules it’s difficult” (Matthew, ON). Some parent participation was required for physical activities, especially if children were younger, an only child, and/or not allowed outdoors alone.

Families faced a number of barriers that made the situation around low PA levels more challenging including inclement weather, motivation, and a lack of opportunities for children to be independently mobile. As Ravi explained, “Until 2 to 3 weeks ago, we couldn’t really go out; It was windy or cold and nowhere to go.” Parents in Ontario, especially, were challenged by a cold and long-lasting winter that seemed to limit many outdoor activities for families. Children’s and parents’ motivation to engage in PA was important in adapting to the pandemic. Dina (ON) explained,

“For me, I’m on the treadmill more. For my kids, it’s more going for a walk for a bike ride, which maybe we’re doing more of because it kinda of feels like it’s the only thing we can do to get outside.”

Reduced options for PA outside and weather were limiting factors for outdoor play opportunities for families.

### 3.4. “I Don’t like It, but It’s a Necessary Evil at This Point”: Pandemic-Related Rises in Screen Time

Parents described a dramatic increase in ST resulting from pandemic-related changes including shifts to online learning for children, reduced access to outdoor destinations for (un)structured activities, socializing with friends by virtual means, and the use of ST as a “’babysitter’. Most parents recounted how their families’ *new normal* was characterized by high levels of ST for their children. As Sarah (ON) described, without having to physically go to school, her children were sitting for,

“five or six hours or even seven hours…So as soon as they get up [in the morning], it starts with YouTube and then cartoons and once the TV is done then they will return to their computer and there has been Google Classroom.”

The time saved by the lack of commuting to school became filled with other ST related pursuits.

Many children transitioned to online school which as Matthew (ON) explained further increased his son’s ST.

“He went from zero during the week [pre-pandemic] to probably four or five hours a day online, including the three, sometimes four hours a day on the weekends. So, I mean, it more than doubled, maybe tripled the amount of screen time he’s getting.”

Parents affirmed that with online school the amount of ST their children engaged in increased. With limited outdoor activities due to harsh weather (especially in Ontario), closed destinations (e.g., playgrounds, pools), or public health restrictions, children tended to engage in more ST. Robert described that his daughter was “on it [screens] more because she was bored” because of the lack of opportunities to do other things outside.

While many parents tried to substitute ST with various activities (e.g., puzzles, art), some children were uninterested. Pritika (ON) explained that her two sons were “not much interested in crafts and puzzles. They used to be into puzzles…but not anymore. But now, both are sitting more yes…it is only screen time.” Other parents explained that their child(ren) were not interested in art, board games, and typically an indoor activity ended up being on screens (e.g., video games, YouTube). While the parents discussed their concern over high ST, they also wanted to recognise the realities of limiting ST during a pandemic. “There has been a little bit more of screen time than before or than what I would like,” Margaret (BC) explained, “but there is only so much you can do as a parent, especially me, where I need to be at my desk during the day.” ST therefore became a part of life during the pandemic because of online school, lack of other activities (e.g., structures activities, destinations to play at, no interest in board games), and time.

A common point of discussion amongst parents was the role ST played as a “babysitter” (Matthew, ON). With many parents working from home, screens became a “necessary evil” as one mother explained (Carmen, BC).

“They actually keep themselves fairly busy that way and kind of leave me alone for the most part, except when they want food… for the most part, they keep themselves pretty occupied with electronics, which is awful. I don’t like it, but it’s a necessary evil at this point.”

Another parent echoed her sentiment, describing his frustration over ST, but when both parents were “dialled into work, there’s nothing we can do about it…sometimes it [screen time] becomes a stop gap for us as a babysitter” (Matthew, ON). Parents recognized the negative effects of ST (e.g., impacts on sleep, sedentary behaviour) and shared their concern about the rising levels of ST, but also acknowledged that it was difficult to limit when parents were also juggling multiple roles as parents, employees, teachers, and/or childcare providers.

Some parents found success with limiting ST on weekends, setting time limits, doing interactive activities (e.g., family walks in the neighbourhood), or other activities (e.g., reading, art). Carmen (BC) explained, “…on the weekends we now make a conscious effort to make sure he’s not getting as much screen time just because during the week it’s kind of a necessity because I have to work...” However, most parents found it challenging to get their children off screens. As Jessie (ON) described, the limiting of ST was a “give or take…You are damned if you do or don’t, if you take everything away from them then what are you left with? Then you can’t get [to] your own stuff.” Many families tried to curb the rising levels of ST for their children, however they encountered various challenges and acknowledged the unprecedented circumstances of living in a pandemic.

Overall, while families expressed concern over rising ST, they also described some benefits. Carmen explained that for her son, “… in this pandemic, [Fortnite] was the only way that he could socialize with his friends” while Margaret explained that video games

“…helped with the 9 weeks he was not able to see anybody. [Her son and his friends] were still able to connect, hear each other voices, even though they were not in the same room they were still playing together.”

ST became the go-to activity with the closures of local destinations like playgrounds, loss of structured activities (e.g., swimming, martial arts), and loss of in-person schooling and socializing.

## 4. Discussion

Our findings extend and contextualize findings from Moore et al. reflecting a dramatic decline in PA and outdoor play among children [3]. While our sampling strategy accounted for provincial differences (ON vs. BC) [5], we could not detect any major differences in parental perceptions besides differences in weather conditions. Families in Ontario discussed the colder conditions and long winter as a major barrier to going outdoors during March and April 2020. Similar to Moore et al., our findings supported that living in a house (e.g., potential for outdoor space) and parents co-participating in physical activities with their children were associated with children’s PA and outdoor activities.

While there is consistency in changes in movement behaviours, the current study provides context to these cross-sectional findings and sheds light on the social-ecological factors influencing those changes. For example, several challenges were discussed by families as they tried to adapt to the pandemic-related restrictions including uncertainty and fear early in the pandemic, the transition to online work and learning, parents juggling multiple roles (e.g., teacher, coach, parent), loss of access to green space (e.g., parks, playgrounds), and public health protocols (e.g., physical distancing). This is in line with recent research demonstrating that where families lived influenced the odds of participating in outdoor activities; for example, children and youth living in houses (compared to apartments) and living further from major roads were more likely to be in the “increased outdoor activities” group compared to the “decreased outdoor activities” group [4]. In addition, parents also described experiencing a number of barriers such as inclement weather (e.g., snow, cold), low motivation (e.g., parent and/or child), and lack of time (e.g., working from home, online school) as being pandemic-related barriers to children’s PA. These barriers are also consistent with barriers to PA among children in general [16].

### 4.1. The Loss of Structured Activity and Children’s Capacity to Move

Our study highlighted an important dilemma faced by families when they lost access to gyms, organized sports, and structured activities (e.g., physical education in schools). Over the last few generations, there has been a shift away from unstructured, routine, organic movement (e.g., independent mobility, free play, unprogrammed sport) toward structured movement and activities (e.g., soccer practice) [17]. In a study examining changes in children’s (8-16 years old) use of time in the United Kingdom between 1975-2015, Mullan found that children spent less time playing outdoors, more time in sport, and more time on screen-based activities [18]. Parental over-reliance on structured activities as a source of PA for their children is an important observation based on the experiences of these parents. This loss was further exacerbated by many children having reduced independent mobility (i.e., a child’s freedom to travel and play within their neighborhood without parental supervision) and reduced opportunity to engage in outdoor play without parental escort [19]. A number of factors can negatively influence independent mobility including individual- (e.g., children at younger ages), social- (perceptions of traffic, unsafe environment), and built environment-factors (e.g., longer distances to destinations) [20]. Supporting children’s independent mobility during the pandemic may be of greater importance now, more than ever. With growing concerns over low levels of children’s PA in the pandemic, independent mobility may be beneficial for PA accumulation [21] besides providing more opportunities for children to be active outdoors without reliance on parents’ schedules. This is particularly relevant, as our findings highlighted that children’s outdoor time was often contingent on parents engineering the opportunity. Some parents expressed the need to supervise their children outdoors due to concerns about adherence to public health protocols (e.g., staying physically distant). With parents trying their best to adapt and to come up with new ways to get their children physically active, independent mobility may be important to support.

### 4.2. Making Good of an Unprecedented Situation

Despite varying public health restrictions, many parents reported similar challenges and adaptations to the COVID-19 pandemic. With many Canadian families grappling with the pandemic-related challenges, it may be important to emphasize the need for self-compassion; which refers to being kind and understanding toward oneself in times of pain or failure [22,23]. Our findings highlighted that families were trying their best in light of public health restrictions, which have the unintended consequences of separating families from friends and loved ones, shifting work and school environments, and limiting destinations and activities families could engage in. With the uncertainty of further waves of the pandemic and the potential for lockdown, challenges for families meeting the recommendations for children’s healthy movement behaviours persist. It is therefore important for public health messaging to acknowledge the challenges families face (e.g., common humanity), help set realistic expectations, and promote supports to assist with addressing challenges.

With the chance of future waves and consequent calls to stay-at-home, it may be prudent to devise evidence-based resources to help families cope with pandemic-related restrictions and support 24-hour movement guidelines for children and youth [1] and adults [24]. Additionally, a re-envisioning of opportunities for PA and reductions in ST is necessary. The findings emphasized that some online physical education and physical activities, much of which were fitness-based (e.g., squats, pushups), were not interesting to children and parents who also raised concern about their children spending more time on screens. Practitioners and researchers need to rethink how to deliver engaging online physical education and activities in addition to providing resources for families that enable healthy movement behaviours. Instead of envisioning a return to what we previously considered *normal*, perhaps this pandemic has emphasized a need to reimagine how children can engage in habitual PA and outdoor play.

### 4.3. Reimagining “Normal”

Previous research highlights the importance of outdoor time for children including accumulation of PA, development of social and motor skills, as well as promoting and maintaining a healthy immune system [25,26]. Our findings highlight a number of social-ecological factors impacting children’s health behaviours. Policy-level factors (e.g., pandemic restrictions) were shown to influence behaviour, as were individual-level (e.g., children’s motivation), interpersonal-level (e.g., parent support, parent work schedules), social-level (e.g., friend availability), built environment-level (e.g., park closures), and natural-level factors (e.g., weather) highlighting the multiple levels of influence of children’s movement behaviours. Perhaps instead of returning to the ‘old’ normal, we instead, reimagine the settings where children play and learn, by creating safe and accessible built environments that support children roaming and playing independently within their neighbourhoods and cities [27,28]. It may be important to develop resources for families to stay healthy and active during pandemic situations—24-hour movement behaviour contingency plans. With increased pandemic-related fear, especially for families with children [29], community support for families in these challenging times is crucial. Key stakeholders including community leaders, policy makers, and health officials need to collaborate on how to support children’s PA and outdoor play [7]. This may include discussions regarding what spaces, such as parks or playgrounds, should be prioritized to remain accessible and what policies would need to be implemented in light potentially ongoing lockdowns.

### 4.4. Strengths and Limitations

To our knowledge, this was the first study to qualitatively examine the impact of the COVID-19 pandemic on Canadian children’s movement behaviours. This method allowed for closer examination of the lived experience of Canadian parents, including identification of common barriers to PA, especially amidst a pandemic, which has been largely unexplored. Such results provide insight on how to shape policy and improve public spaces to promote PA now and in the future. The sample was focused on urban areas in two major cities in British Columbia and Ontario and half the participants had a household income over $100,000. Additionally, participants indicated their interest in participating in interviews, which may introduce self-selection bias; participants may have had a particular interest or concern about movement behaviours which was different to those we did not interview. Future research may need to examine families’ pandemic experiences in suburban and rural locations as well as in disadvantaged neighbourhoods where circumstances may be different (e.g., no outdoor space, less population density) and there may be different barriers to meeting healthy movement behaviour guidelines.

## 5. Conclusions

The COVID-19 pandemic has had a profound impact on Canadian families and children’s movement behaviours. Families are trying to cope but are faced with a number of challenges including constantly changing public health restrictions, parents juggling multiple roles, online school, and weather. It remains important to continue to encourage time outdoors, to support policy and practice initiatives supporting childhood independent mobility, and to develop an inventory of family-oriented resources that can help families achieve and maintain healthy movement behaviours [7]. Future research is needed exploring how Canadian families are adapting over the long term to these challenging circumstances.

## Figures and Tables

**Table 1:** Participant demographics.

Demographics	*n*	%
**Age**	42.9 ± 4.64	
**Gender**		
Woman	10	34.5
Man	19	65.5
**Ethnicity**		
Aboriginal (e.g., Inuit, Métis, North American Indian)	2	6.9
African (e.g., South African, Ethiopian, Nigerian, Zimbabwean)	1	3.4
Arab/West Asian (e.g., Lebanese, Moroccan, Iranian, Turk)	1	3.4
British Isles (e.g., English, Irish, Scottish)	9	31.0
Chinese	3	10.3
French	1	3.4
Jewish (non-denominational)	1	3.4
South Asian (e.g., East Indian, Pakistani, Goan, Sri Lankan)	9	31.0
Other East and Southeast Asian (e.g., Filipino, Vietnamese, Korean, Japanese)	2	6.9
Other European (e.g., German, Russian, Italian, Norwegian)	5	17.2
Other North American (e.g., Canadian, American, Newfoundlander, Québécois)	5	17.2
Other	1	3.4
**Marital Status**		
Common Law	1	3.4
Married	24	82.8
Divorced	4	13.8
**Employment**		
Employed/Self-employed full-time (30+ hours/week)	22	75.9
Employed/Self-employed part-time (<30 hours/week)	3	10.3
Homemaker	3	10.3
Currently looking for work	1	3.5
**Income**		
$35,000–75,000	8	27.6
$75,000–125,000	8	27.6
$125,000–250,000	12	41.4
Undisclosed	1	3.4
**Education**		
Some college/Technical school	7	24.1
Completed college	5	17.2
Some university	1	3.5
University undergraduate degree	7	24.1
Some post-graduate school	2	6.9
Post-graduate degree	7	24.1
Child Age (years)		
5–6	8	27.6
7–9	9	31.0
10–11	12	41.4
**Child Gender**		
Girl	13	44.8
Boy	16	55.2

## Data Availability

The data are not publicly available due to confidentiality concerns.

## Supplementary Materials

The following are available online at https://www.mdpi.com/2227-9067/8/3/219/s1, File S1: Children & Youth Movement and Play Behaviours Survey: Impact of the 2020 COVID-19 Outbreak Interview Guide.
