# Supplementary material for: “You Can’t Go to the Park, You Can’t Go Here, You Can’t Go There”: Exploring Parental Experiences of COVID-19 and Its Impact on Their Children’s Movement Behaviours
Source: Children (Basel). 2021 Mar 12;8(3):219. doi: 10.3390/children8030219 (PMC8000735; doi:10.3390/children8030219)
Supplement: Supplementary file 1 [file children-08-00219-s001.pdf]

Supplemental File S1

CHILDREN & YOUTH MOVEMENT AND PLAY BEHAVIOURS SURVEY:

IMPACT OF THE 2020 COVID-19 OUTBREAK INTERVIEW GUIDE

The World Health Organization (WHO) declared COVID-19 as a pandemic on March 11, 2020. For the following questions, we would like you to consider your child's movement behaviours (physical activity, sedentary behaviours and sleep) during the first three months of the COVID-19 related restrictions.

Setting the context

1. Can you describe the pandemic-related restrictions your family has experienced where you live?
   - *Prompts*: what were you allowed to do; what were you not allowed to do?
2. When, if at all, did the restrictions feel most challenging?
   - Prompts: weekdays versus weekends? Morning/afternoons? Rainy/sunny days?
3. At that time, how did these restrictions affect your family?
   - *Prompts*: did things change over time? How do they feel now?

Changes in movement behaviours

The next questions are about your child. If you have more than one child, please think about the one whose first name is first in the alphabet.

4. Let's think about a typical day when you felt the restrictions were most challenging. Can you walk me through what that day was like for your family? For your child?
   - *Prompts:* changes in...
     - Sleep duration? Sleep quality?
     - Sleep routines (e.g., going to bed/waking up same time)?
   - *Prompts*: did time spent playing outdoors...
     - Increase, decrease, stay the same?
     - In what way? How has it changed? Why?
     - Was your child allowed outdoors without you? Has this changed (distance, who with)?
     - Do you feel any social pressure or encouragement to keep your child(ren) inside? In what way?
     - Have any concerns you may have had about your child being outdoors changed?
   - *Prompts*: moderate-to-vigorous physical activity (activity that made your child out of breath) has...
     - Increased, decreased, stayed the same?
     - In what way? How has it changed (e.g., indoors/outdoors; who with)? Why?
   - *Prompts:* screen time has...
     - Increased, decreased, stayed the same?
     - In what way? How has it changed (e.g., who with)? Why?
   - *Prompts*: other sedentary behaviours like reading, crafts, and puzzles have...
     - Increased, decreased, stayed the same?
     - In what way? How has it changed (e.g., indoors/outdoors, who with)? Why?

5. Do you think your neighbourhood built environment (such as buildings, roads, access to parks) is a barrier to your child being outdoors during the times of restriction? In what ways?
6. What impact have these changes had on your family? How you interact with your child (e.g., spend more time with your child)? Yourself (e.g., got more active)? Your child (e.g., changes in fitness?
7. Overall, how has your family adapted to the pandemic in relation to movement behaviours?
8. Compared to other families like yours, how well do you think you and your family have adapted to the pandemic in terms of movement behaviours?

Changes in support behaviours

9. Have there been any changes in how you support your child in engaging in healthy movement behaviours during the pandemic-related restrictions?
    - *Prompts*:
        - Encouragement (play, physical activity, sleep)?
        - Co-participation (play, physical activity)?
        - Setting screen time restrictions?
        - Anything that you do differently to support your child?
10. What has worked in supporting your child to engage in healthy movement behaviours?
11. What barriers have you faced in supporting your child to engage in healthy movement behaviours? How have you managed these?
    - Prompts: did you feel you could support (capability) but not worth the hassle (motivation)?

Looking ahead

12. Having experienced the impact of physical distancing and other related restrictions, is there anything that would have helped you in supporting your child's movement behaviours?
    - *Prompts*:
        - What has helped the most?
        - Anything you wished you had known?
        - Any resources that you wished you had?
        - What would help you now?
13. There is now talk about loosening restrictions and opening things up again. How will things change, if at all, for your child in terms of their outdoor play, physical activity and screen time?
    - *Prompts*: Return to pre-pandemic levels? What concerns might you have about transitioning to the 'new normal'?
14. Do you have any **advice for *other* families** like yours trying to achieve a healthy balance of movement behaviours (physical activity, sedentary behaviours, sleep) for their children during the COVID-19 pandemic and related restrictions?
